# Microtubule associated protein 4 phosphorylation-induced epithelial-to-mesenchymal transition of podocyte leads to proteinuria in diabetic nephropathy

**DOI:** 10.1186/s12964-022-00883-7

**Published:** 2022-07-28

**Authors:** Lingfei Li, Yanhai Feng, Junhui Zhang, Qiong Zhang, Jun Ren, Cheng Sun, Shujing Li, Xia Lei, Gaoxing Luo, Jiongyu Hu, Yuesheng Huang

**Affiliations:** 1 Department of Dermatology, Daping Hospital, Third Military Medical University (Army Medical University), Chongqing, China; 2 Institute of Burn Research, Southwest Hospital, Third Military Medical University (Army Medical University), Chongqing, China; 3 State Key Laboratory of Trauma, Burns and Combined Injury, Southwest Hospital, Third Military Medical University (Army Medical University), Chongqing, China; 4 Department of Cardiology, Shanghai Institute of Cardiovascular Diseases, Zhongshan Hospital, Fudan University, Shanghai, 200032 China; 5 Department of Laboratory Medicine and Pathology, University of Washington, Seattle, WA 98195 USA; 6 Department of Ophthalmology, Southwest Hospital, Third Military Medical University (Army Medical University), Chongqing, China; 7 The Second Affiliated Hospital of Chongqing Medical University, Chongqing, China; 8 Endocrinology Department, Southwest Hospital, Third Military Medical University (Army Medical University), Chongqing, China; 9 Department of Wound Repair, Institute of Wound Repair and Regeneration Medicine, Southern University of Science and Technology Hospital, Southern University of Science and Technology School of Medicine, Shenzhen, China

**Keywords:** MAP4, Diabetic nephropathy, Proteinuria, Epithelial-to-mesenchymal transition, Apoptosis

## Abstract

**Background:**

Diabetic nephropathy (DN) involves various structural and functional changes because of chronic glycemic assault and kidney failure. Proteinuria is an early clinical manifestation of DN, but the associated pathogenesis remains elusive. This study aimed to investigate the role of microtubule associated protein 4 (MAP4) phosphorylation (p-MAP4) in proteinuria in DN and its possible mechanisms.

**Methods:**

In this study, the urine samples of diabetic patients and kidney tissues of streptozotocin (STZ)-induced diabetic mice were obtained to detect changes of p-MAP4. A murine model of hyperphosphorylated MAP4 was established to examine the effect of MAP4 phosphorylation in DN. Podocyte was applied to explore changes of kidney phenotypes and potential mechanisms with multiple methods.

**Results:**

Our results demonstrated elevated content of p-MAP4 in diabetic patients’ urine samples, and increased kidney p-MAP4 in streptozocin (STZ)-induced diabetic mice. Moreover, p-MAP4 triggered proteinuria with aging in mice, and induced epithelial-to-mesenchymal transition (EMT) and apoptosis in podocytes. Additionally, p-MAP4 mice were much more susceptible to STZ treatment and showed robust DN pathology as compared to wild-type mice. In vitro study revealed high glucose (HG) triggered elevation of p-MAP4, rearrangement of microtubules and F-actin filaments with enhanced cell permeability, accompanied with dedifferentiation and apoptosis of podocytes. These effects were significantly reinforced by MAP4 hyperphosphorylation, and were rectified by MAP4 dephosphorylation. Notably, pretreatment of p38/MAPK inhibitor SB203580 reinstated all HG-induced pathological alterations.

**Conclusions:**

The findings indicated a novel role for p-MAP4 in causing proteinuria in DN. Our results indicated the therapeutic potential of MAP4 in protecting against proteinuria and related diseases.

**Video Abstract**

**Supplementary Information:**

The online version contains supplementary material available at 10.1186/s12964-022-00883-7.

## Background

Diabetic nephropathy (DN) is the most common form of complication for renal replacement therapy and prompts end-stage renal failure in diabetes mellitus [14, 28]. DN involves a series of structural and functional changes when subjected to chronic glycemic assault [4]. Profound proteinuria, which heralds a gradual deterioration in glomerular function, commonly occurs in the early stage of DN [38]. The magnitude of proteinuria has been commonly employed to assess DN severity. Despite considerable advances in understanding the mechanisms governing the permselective properties of glomerular capillary wall, pathogenesis of proteinuria remains unclear in DN.

Ample evidence has depicted that podocyte slit diaphragm and foot processes are important barriers of glomerular filter, and breakage of the barrier integrity is a leading cause in the progression of proteinuria [31]. Among various available pathogenic theories for podocyte dysfunction in DN, epithelial-to-mesenchymal transition (EMT) is one of the responses that occurs in certain kidney diseases [20]. When subjected to EMT, cytoskeleton of podocytes rearranged, destroyed their cell junctions and basal–apical polarity, altered their interaction with extracellular matrix, and obtained mesenchymal features, such as invasiveness and elevated motility [36].

Microtubule-associated proteins (MAPs) are primarily recognized as skeletal proteins which bind to tubulins to promote their assembly. The family of MAPs encompass tau, MAP2 and MAP4. MAP4 is widely expressed in non-neural cells and plays an essential role in maintaining microtubule (MT) dynamics [8, 17, 19, 23]. Additionally, MT-MAPs binding is modulated by MAPs phosphorylation which leads to dissociation of MAPs from MT and instability of MT [10, 16]. We and others have reported that the human MAP4 (S768 and S787) phosphorylation is crucial in governing its detachment from MTs, and mitogen-activated protein kinase 6 (MKK6)/p38 MAPK serves as the upstream signal for hypoxia-mediated MAP4 phosphorylation and followed by MT depolymerization [17, 21, 30].

In current study, we firstly demonstrated increased MAP4 phosphorylation in the urine samples of diabetic patients and the kidneys of streptozotocin (STZ)-induced diabetic mice. Subsequently, we constructed mouse models to mimic MAP4 phosphorylation in specific-sites (S737 and S760). Somewhat to our surprise, MAP4 phosphorylation initiated proteinuria with aging, accompanied with incremental EMT and apoptosis of podocytes. In addition, MAP4 phosphorylation mice were much more susceptible to STZ treatment and showed robust DN pathology as compared to wild-type mice. In vitro, F-actin and MT rearrangement induced by MAP4 phosphorylation was deemed potential mechanisms of podocyte EMT, promoting to the development of proteinuria. Altogether, the findings favor a novel role for MAP4 phosphorylation in contributing to proteinuria in DN, indicating its therapeutic potential in the management of proteinuria and related diseases.

## Materials and methods

### Ethics statement of patient urine samples

The Ethics Committee of Southwest Hospital, Third Military Medical University (Army Medical University) approved the present study (approval number: KY2020085). Prior to be included in the study, all participants signed relevant informed consent. Participants were identified using a number, not their name. It was conducted in adherence with the Declaration of Helsinki. All methods were carried out in accordance with the institutional guidelines of the ethics committee at Southwest Hospital, Third Military Medical University (Army Medical University) (Chongqing, China).

### Urine sample collection

Eight urine samples of patients with clinically diagnosed diabetes were collected from the inpatients who signed a consent form. These inpatients received formal treatment at Endocrinology Department, Southwest Hospital, Third Military Medical University (Army Medical University). The samples were collected for three consecutive days, and the samples used were the urine left over from routine urine collections, which were midstream samples. All samples were assigned a study number that connected them to clinical information from the chart to identify them. The samples were used for ELISA analysis and the data were averaged.

### Ethics statement of animal study

All animal experiments were implemented in accordance with the Guide for the Care and Use of Laboratory Animals announced by the US National Institutes of Health (NIH Publication, 8th Edition, 2011) and were granted permission by the Animal Experiment Ethics Committee of the Army Medical University (the Third Military Medical University).

### Animal study

The knock-in mice that mimicked MAP4 hyperphosphorylation in specific-sites (S737 and S760; MAP4 KI mice) were performed as previously described [24]. Wild-type (WT) littermates were used as controls for experiments with corresponded MAP4 KI male or female mice. The age of mice ranged from 10 to 74 weeks. The C57BL/6J or MAP4 KI mice (10–74 weeks) applied for STZ (Cat# S0130, Sigma, Darmstadt, Germany)-induced diabetes were starved for 8–12 h and then injected with a single intraperitoneal dose of STZ in sodium citrate buffer at 150 mg/kg body weight. Mice with a glucose concentration exceeding 16.7 mmol/L were considered diabetic. Mice were anesthetized with pentobarbital sodium (1%, 50 mg/kg) by intraperitoneal injection and sacrificed 5 weeks after STZ injection. Experiments involving animals were performed in accordance with United Kingdom Home Office and European Union guidelines and were approved by the Animal Care Centre of the Third Military Medical University. The detailed information about the number of mice was described as follows. WT 10-14w 26 males and 26 females, WT 30-34w 13 males and 13 females, WT 70-74w 13 males and 13 females; WT STZ 17 males and 17 females; MAP4 KI 10-14w 16 males and 16 females, MAP4 KI 30-34w 13 males and 13 females, MAP4 KI 70-74w 13 females and 13 males; MAP4 KI STZ 7 males and 7 females.

### Biochemical tests of urine and blood samples

Mouse random urine was collected and centrifuged, and blood samples were collected by retro-orbital puncture from anesthetized animals after fasting 8–12 h. Urine albumin, urine creatinine (CR), serum levels of urea nitrogen (UN)and cystatin c (Cys-c) were determined using commercial kits. The detailed information of commercial kits is as follows: Urine albumin (Cat.#447690, Beckman coulter, USA), urine creatinine (CR) (Cat.#1.02.4102, Fosun Diagnostics, China), urea nitrogen (UN) (Cat.#OSR6134, Beckman coulter, USA) and cystatin c (Cys-c) (Cat.#H419T3, MedicalSyatem, China).

### Histology and ultrastructural pathology

Renal tissue was cut and fixed with 10% formalin, embedded in paraffin or made in frozen, and sectioned at 5-μm thickness for routine histopathology or Masson’s trichrome staining. Six serial sections from each mouse were used to measure the glomerular volume: the volume of the glomerular was calculated as previously described [3, 29]. For transmission electron microscope (TEM) observation, tissues of renal cortical were fixed in 2.5% glutaraldehyde followed by dehydration, vibratome sliced and recut on a microtome and stained with uranyl acetate and lead citrate overnight. The degree of foot process fusion was determined by measuring the number of junctions per 1 micron length of glomerular basement membrane (GBM) as described previously [1].

### Podocyte culture and treatment

Conditioned immortalized murine podocytes were obtained and cultured as our study described previously [3, 33]. Cells were used for experiments as stated. After transfection of adenovirus overexpressing MAP4(Ala), MAP4(Glu), MKK6(Glu), or CMV-null for 36 h, the cells were treated with 5.6 mM d-glucose, 5.6 mM d-glucose + 25 mM mannitol or 30 mM d-glucose for 48 h. During the stimulation, SB203580 (Calbiochem Cat# 559389, 10 μM, Darmstadt, Germany) was applied to pretreat cells for 1 h prior to glucose treatment.

### Adenovirus construction

Site-directed mutagenesis of MAP4 and MKK6, construction and transduction of recombinant adenoviruses overexpression MAP4(Glu), MAP4(Ala) and MKK6(Glu) were performed as described previous [16, 23, 24]. MAP4(Ala) mimicked MAP4 (S737 and S760) dephosphorylation, MAP4(Glu) mimicked MAP4 (S737 and S760) phosphorylation and MKK6(Glu) was an upstream kinase of p38/MAPK, which was consistently activated p38/MAPK.

### Immunofluorescence

Podocytes or frozen sections were used as described previously in our reports [24]. Antibodies: α-tubulin (Proteintech Group Cat# 11224-1-AP, RRID:AB_2210206, Rosemont, IL, USA), Phalloidin (Thermo Fisher Scientific Cat# A12379, RRID:AB_2315147, Waltham, MA, USA), Wilms Tumor protein (WT-1) (Abcam Cat# ab89901, RRID:AB_2043201, Cambridge, Cambridgeshire, United Kingdom), fibroblast-specific protein 1 (FSP1) (Abcam Cat# ab197896, RRID:AB_2728774), Nephrin (R and D Systems Cat# AF3159, RRID:AB_2155023, Minneapolis, MN, USA), Desmin (R and D Systems Cat# AF3844, RRID:AB_2092419).

### Apoptosis assay

In Situ Cell Death Detection Kit, fluorescein (Cat# 11684795910, Roche, Basel, Switzerland) were used for apoptosis assay of frozen kidney sections (WT-1 and terminal deoxynucleotidyl transferase-mediated dUTP nick-end labeling (TUNEL) co-staining) or podocytes.

### Cell proliferation assay

Cell proliferation determined by 5-ethynyl-2'-deoxyuridine (EdU) assay was carried out using the Click-iT® EdU imaging detecting kit according to the manufacturer's instructions (Cat# BCK488-IV-IM-S, Sigma).

### Western blot (WB) analysis

Renal tissue samples and podocytes were used for WB experiments following our previously described procedures [24]. Antibodies: WT-1 (Abcam Cat# ab89901, RRID:AB_2043201), FSP1 (Abcam Cat# ab197896, RRID:AB_2728774), Nephrin (R and D Systems Cat# AF3159, RRID:AB_2155023), Desmin (R and D Systems Cat# AF3844, RRID:AB_2092419), GAPDH (Proteintech Group Cat# 60004-1-Ig, RRID:AB_2107436), MAP4 (Bethyl Cat# A301-489A, RRID:AB_999616, Montgomery, Texas, USA), p38 MAPK (P38) (Cell Signaling Technology Cat# 9212, RRID:AB_330713, Danvers, MA, USA), Phospho-p38 MAPK (p-P38) (Cell Signaling Technology Cat# 9211, RRID:AB_331641). Rabbit polyclonal antibodies against p-MAP4 (S787) and p-MAP4 (S737) were made in-house and validated as reported previously [24].

### Enzyme-linked immunosorbent assay (ELISA)

Specific ELISA kits were purchased from the Mlbio Group to detect the concentrations of MAP4 (Cat. #ml211307-C, Mlbio, China)and p-MAP4 (Cat. #ml221799-C, Mlbio, China) in urine samples. Detection procedures were conducted according to the manufacturer’s instructions.

### Measurement of transepithelial electric resistance (TER)

Cellular barrier properties were assessed using assessment of TER across confluent podocytes with Millicell-ERS (MERS00002, Millipore, Darmstadt, Germany) [23]. A 24-well transwell system (Greiner Bio-One, 0.4-mm pore size, 6.5-mm diameter, transparent, Costar) was inserted into the 24-well plate. Podocytes were plated in transwell chambers. The volumes of culture medium were 100 and 600 μL in the upper and lower compartments, respectively, in which cells were allowed to grow for 2 days (considered day 0). The resistance (R sample) was measured; the value for the blank well represented the blank resistance (R blank). The following calculation was performed: epithelial monolayer resistance = (R sample-R blank) * effective membrane area (the area of a 24-well transwell chamber is 0.336 cm^2^).

### Measurement of the permeability to fluorescein isothiocyanate (FITC)-dextran

Cells were grown on transwell compartments as above described. The permeability was measured by the addition of FITC-dextran (40 kDa; Sigma-Aldrich) for 1 h [23].

### Retinal angiography imaging

Angiography imaging was obtained with spectralis HRA + OCT (Heidelberg Engineering, Heidelberg, Germany). In brief, pupils were dilated using topical tropicamide phenylephrine eye drop (Mydrin-P, Santen. Oy, Japan). Mice were anesthetized as described above. Fluorescein sodium salt (5 ml:0.5 g; Alcon Laboratories, Freiburg, Germany) was injected intraperitoneally as fluorescent tracer. Imaging was obtained at 488-nm absorption and 495-nm emission using a 55° lens. Images of the central retina were taken, with the optic nerve located in the center of the image.

### Statistical analysis

All values are means ± SEM (for bar graphs) or SD (for univariate scatter plots). Statistical differences between groups were assessed using two-tailed Student's *t* test or one-way analysis of variance (ANOVA) post hoc tests, as appropriate. For all studies, values of *P* < 0.05 were considered statistically significant.

## Results

### MAP4 phosphorylation increased in urine samples of diabetic patients

**Fig. 1:**
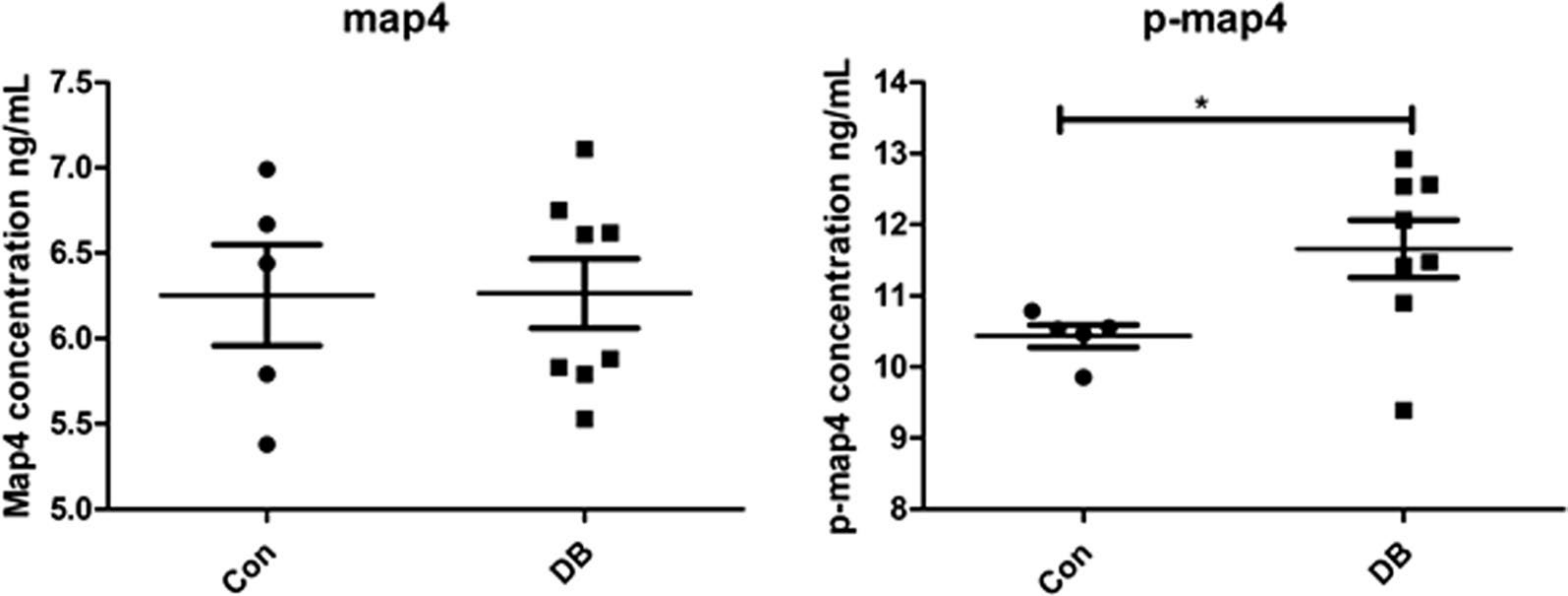
MAP4 phosphorylation increased in urine samples of diabetic patients. Concentrations of MAP4 (**A**) and p-MAP4 (**B**) in urine samples were measured by ELISA. n = 5 or 8. *P < 0.05 when compared with control group. *P* value were derived from two-tailed Student's *t*-test

To identify the variations of MAP4 and its phosphorylation in patients with clinically diagnosed diabetes, we measured them through ELISA after urine of diabetic patients was collected. As indicated in Fig. 1, a significant up-regulation of MAP4 phosphorylation was observed in urine (*p* < 0.05), and simultaneously, no obvious variation of total MAP4 protein was noted (*p* > 0.05). This result demonstrated that increased MAP4 phosphorylation existed in urine of diabetic patients.

### Nephritic MAP4 hyperphosphorylation along with proteinuria in DN

**Fig. 2:**
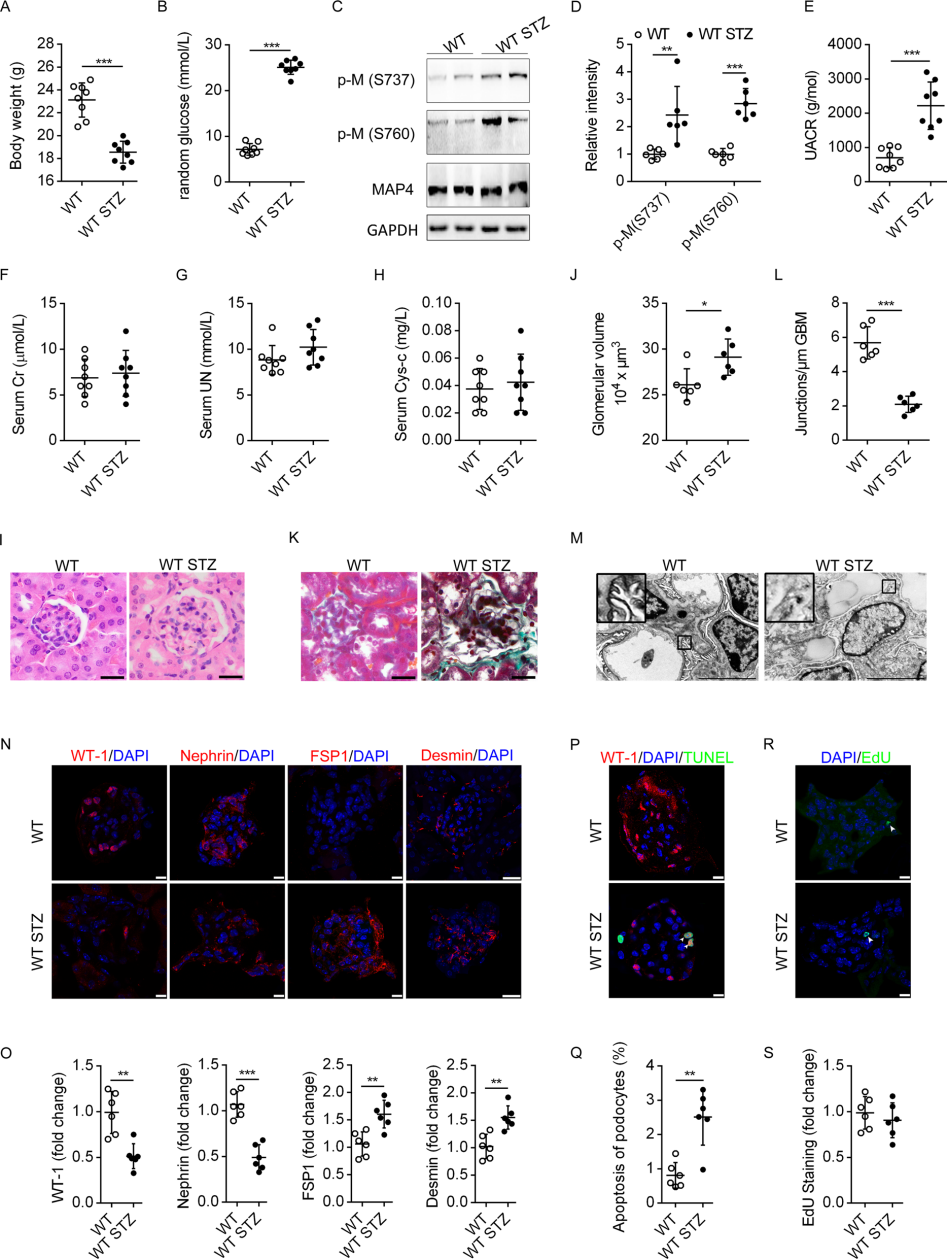
Nephritic MAP4 hyperphosphorylation along with proteinuria in DN. **A**, **B** Body weight (**A**) and random blood glucose (**B**) was assessed between WT and WT STZ mice. n = 8. **C**, **D** Representative WB (**C**) and quantitative analysis (**D**) depicting p-M (S737 and S760) in WT and WT STZ littermates. n = 6. p-M, p-MAP4. **E–H** UACR, serum levels of CR, UN and Cys-c were detected by commercial kits. n = 8. **I**, **J** HE staining and glomerular volume quantitative analysis of deparaffinized kidney tissue section between WT and WT STZ mice. Bar, 20 µm. n = 6. **K** Masson’s trichrome staining of WT and WT STZ mice. Bar, 20 µm. n = 6. **L**, **M** Quantitative analysis of foot processes fusion (**L**) and TEM observation (**M**). Bar, 5 µm. n = 6. **N**, **O** Representative confocal immunofluorescence images and quantitative analysis showing the epithelial and mesenchymal cell markers of frozen kidney tissue section. Bar, 10 or 25 µm. n = 6. **P**, **Q** Podocyte apoptosis (white arrows) of frozen kidney tissue section was detected by WT-1 and TUNEL co-staining. Bar, 10 µm. n = 6. **R**, **S** Cell proliferation (white arrows) of frozen kidney tissue section was determined by EdU staining. Bar, 10 µm. n = 6. The graph showed mean ± SD. *P < 0.05, **P < 0.01, ***P < 0.001. *P* values were derived from two-tailed Student's *t*-test

A significant reduction in body weight and random blood glucose were observed in STZ experimental diabetic mice compared with the WT littermates (Fig. 2A, B). Using WB analysis, phosphorylation levels of MAP4 at S737 and S760 were significantly elevated in renal tissues from STZ-induced diabetic mice compared with WT mice (Fig. 2C, D). Although little changes were noted in serum CR, serum UN and serum Cys-c between the two groups (Fig. 2F–H), the early indicator of renal injury-urine albumin/CR ratio (UACR) was significantly elevated in STZ-induced diabetic mice (Fig. 2E). An increase in glomerular volume was evident using the haematoxylin–eosin (HE) staining (Fig. 2I, J) and Masson’s trichrome staining displayed basement membrane thickening, collagen accumulation and a rise in mesangial matrix (Fig. 2K). Ultrastructure of glomerular filtration barrier was further examined using TEM. STZ treatment induced foot process fusion (as depicted in enlarged view in Fig. 2M), associated with severely damaged cytoplasmic structures of podocytes (Fig. 2M). To assess the degree of foot process fusion, the number of junctions per μm GBM was determined (Fig. 2L). WT mice possess much more junctions per μm GBM than STZ mice, suggesting enhanced foot processes effacement in STZ mice. We further examined the effect of STZ on epithelial and mesenchymal markers in podocytes. As shown in Fig. 2N, O immunofluorescence staining revealed that STZ challenge inhibited the levels of epithelial markers, including Nephrin and WT-1, while promoting mesenchymal markers expression, including FSP-1 and Desmin. In addition, WT-1, a marker of podocyte, and TUNEL co-staining revealed increased podocyte apoptosis (white arrows) in glomeruli (Fig. 2P, Q). Little difference was noted in cell proliferation using EdU staining between STZ and WT group (Fig. 2R, S). Taken together, these data indicated that STZ treatment led to overt albuminuria, podocyte dedifferentiation and apoptosis in glomeruli.

### MAP4 hyperphosphorylation mutation leads to proteinuria and nephropathy reminiscent of diabetic kidney disease in MAP4 KI mice with or without STZ stimulation

**Fig. 3:**
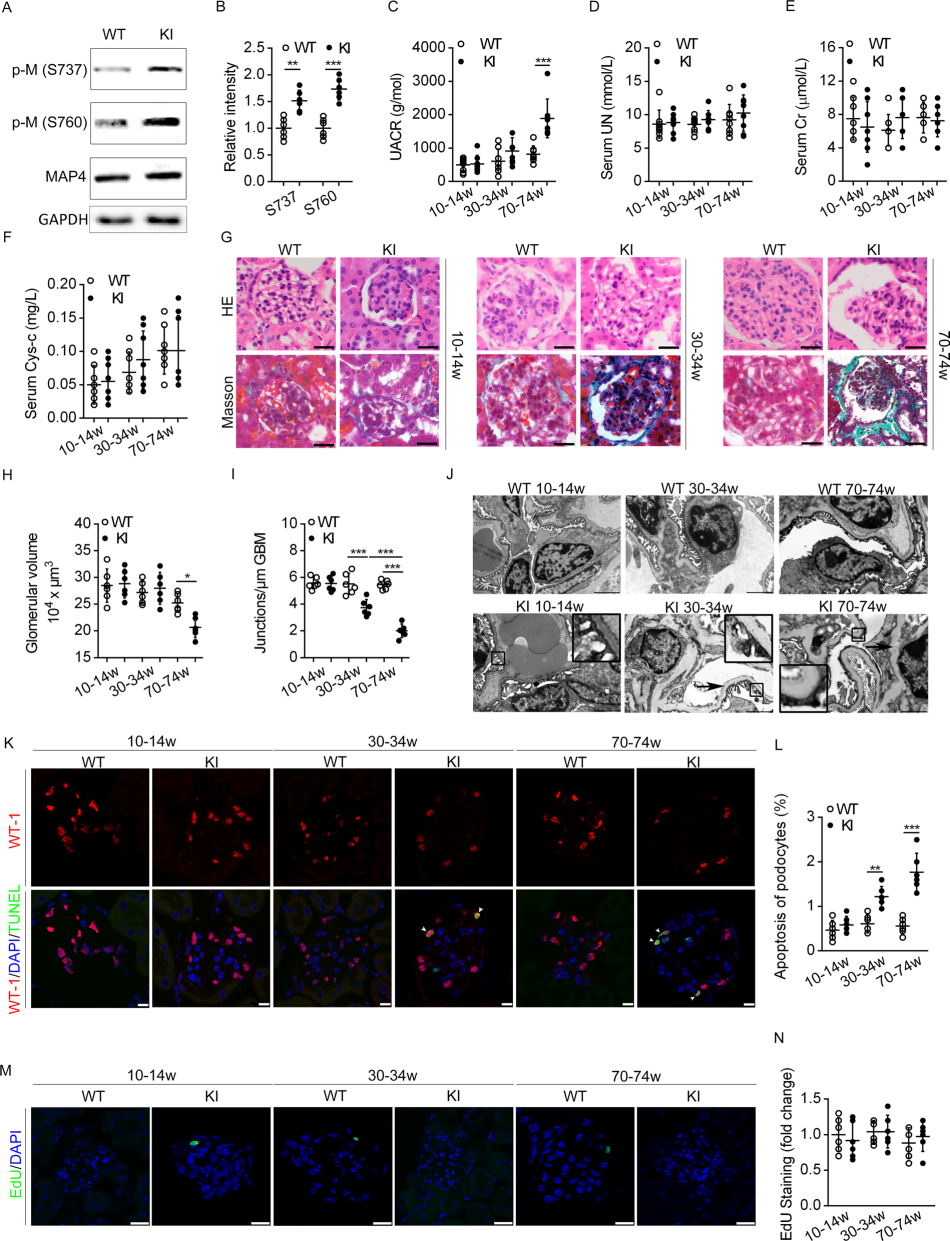
MAP4 hyperphosphorylation mutation leads to proteinuria and nephropathy reminiscent of diabetic kidney disease in MAP4 KI mice. **A**, **B** Representative WB (**A**) and quantitative analysis (**B**) depicting p-M (S737 and S760) in WT and MAP4 KI mice at 10–14 weeks. n = 6. **C–F** UACR, serum levels of CR, UN and Cys-c were determined in two mouse models at different ages. n = 8. **G**, **H** HE and Masson’s trichrome staining as well as glomerular volume quantitative analysis of deparaffinized kidney tissue section was examined at different ages. Bar, 20 µm. n = 6. **I**, **J** Quantitative analysis of foot processes fusion (**I**) and TEM observation (**J**). Bar, 2 µm. n = 6. **K**, **L** Podocyte apoptosis of frozen kidney tissue section was detected by WT-1 and TUNEL co-staining. Bar, 10 µm. n = 6. **M**, **N** Cell proliferation of frozen kidney tissue section was determined by EdU staining. Bar, 25 µm. n = 6. The graph showed mean ± SD. *P < 0.05, **P < 0.01, ***P < 0.001. *P* values were derived from one-way ANOVA with Bonferroni’s post-test

**Fig. 4:**
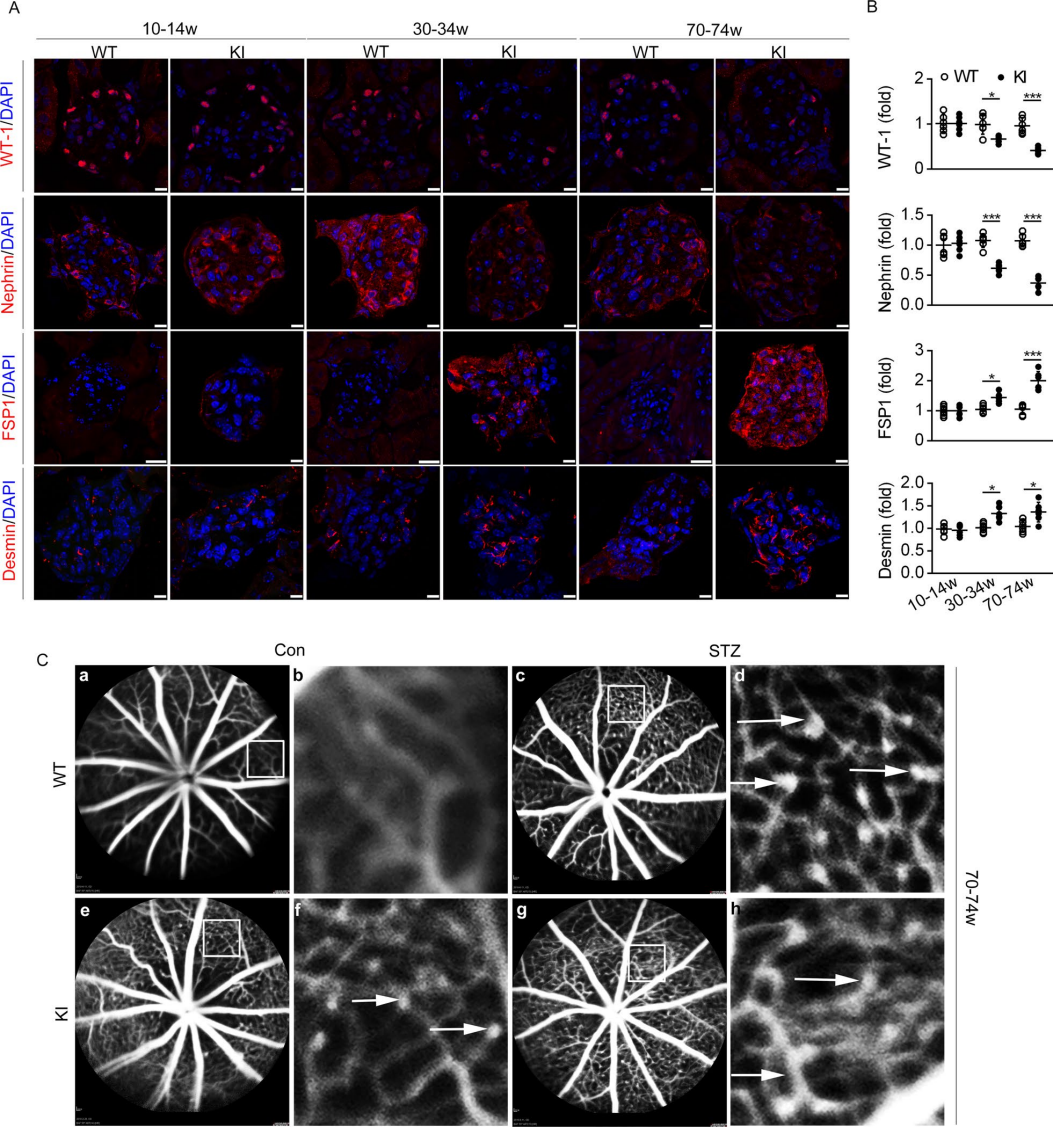
MAP4 hyperphosphorylation mutation leads to EMT and diabetic retinopathy in MAP4 KI mice. **A**, **B** Representative confocal immunofluorescence images (**A**) and quantitative analysis (**B**) showing the epithelial and mesenchymal cell markers of frozen kidney section at different ages. Bar, 10 or 25 µm. n = 6. **C** Fluorescein angiogram showing retinal vasculature of (a) normal control mouse (c) STZ-induced diabetic mouse (e) MAP4 KI mouse (g) STZ treated MAP4 KI mouse. The (b), (d), (f) and (h) are the represented enlarged area of the (a), (c), (e) and (g). Arrow points to the microaneurysms. *P < 0.05, ***P < 0.001.* P* values were derived from one-way ANOVA with Bonferroni’s post-test

To elucidate potential pathological role of MAP4 phosphorylation in kidney disease, renal function and morphology were examined in MAP4 KI mice. MAP4 KI mice showed increased phosphorylation levels of MAP4 at S737 and S760 in renal tissues compared with those from WT normal mice (Fig. 3A, B). Little changes in serum levels were noted for CR, UN and Cys-c between the two groups (up to 70-74w) (Fig. 3D–F), while UACR was drastically elevated in MAP4 KI mice at 70–74 weeks of age (Fig. 3C). Interestingly, a decrease in volume of glomerular volume in MAP4 KI mice was detected at the age of 70–74 weeks using HE staining (Fig. 3G, H). An increase in basement membrane thickening and collagen accumulation was observed by Masson Trichrome staining at the ages of 30-34w (Fig. 3G). Using TEM evaluation, widening of slit membranes was detected in podocytes (enlarged view in Fig. 3J) and WT mice displayed much more junctions per μm GBM compared with MAP4 KI mice at the age of 30–34 and 70–74 weeks (Fig. 3I), suggesting increased foot processes effacement in MAP4 KI mice. Profound thickening of basement membrane and podocytes detachment were also seen in MAP4 KI mice at the age of 30–34 and 70–74 weeks (black arrowheads in Fig. 3J). Co-staining of WT-1 and TUNEL revealed elevated apoptosis of podocytes (white arrowheads) at 30–34 and 70–74 weeks of age in MAP4 KI mice as compared to WT littermates (Fig. 3K, L). There was little difference between MAP4 KI and WT groups in cell proliferation using EdU staining (Fig. 3M, N). Immunofluorescence staining revealed decreased expressions of epithelial markers, such as Nephrin and WT-1, and elevated expressions of mesenchymal indicators, including FSP1 and Desmin in glomeruli of 30–34 and 70–74 week-old MAP4 KI mice, indicating podocyte dedifferentiation (Fig. 4A, B). Given that retinopathy is an important microvascular complication of diabetes, concurrent with DN, we further investigated retinopathy in MAP4 KI mice. Our results revealed normal retinal vasculature in WT mice at 70–74 weeks of age (Fig. 4C a, b). The data shown in Fig. 4C e–f exhibited microscopic aneurysms with balloon-like structures budding off capillary walls in retinal vessels from 70-74w mutant mice, similar to STZ-induced diabetic model (Fig. 4C c, d). MAP4 KI aggravated STZ-induced microscopic aneurysms (Fig. 4C g, h).

**Fig. 5:**
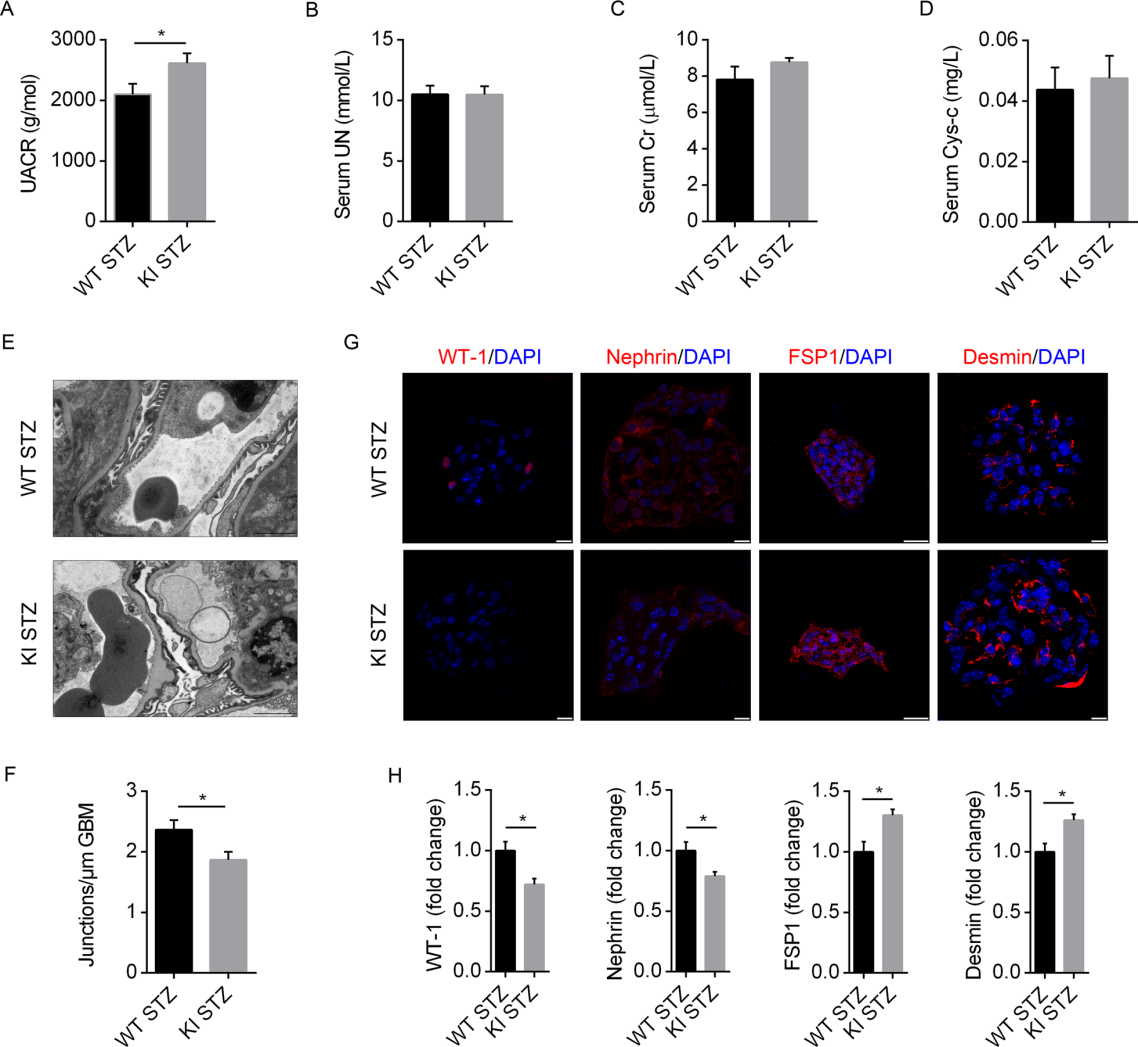
Detection of proteinuria and nephropathy reminiscent of diabetic kidney disease in STZ-induced WT and MAP4 KI mice. **A–D** UACR, serum levels of CR, UN and Cys-c were determined in two mouse models at 10–14 weeks after STZ treatment. n = 8. **E**, **F** Quantitative analysis of foot processes fusion and TEM observation in WT and MAP4 KI mice at 10–14 weeks after STZ treatment. Bar, 2 µm. n = 6. **G**, **H** Representative confocal immunofluorescence images and quantitative analysis showing the epithelial and mesenchymal cell markers of frozen kidney section in two mouse models at 10–14 weeks after STZ treatment. Bar, 10 or 25 µm. n = 6. Data were shown as mean ± SEM. *P < 0.05.* P* values were derived from two-tailed Student's *t*-test

To detect the kidney phenotypes of WT and MAP4 KI mice after STZ treatment, renal function and morphology were further examined at the age of 10–14 weeks between the two mice. The results demonstrated that MAP4 KI STZ mice displayed deteriorated pathological kidney changes as compared to WT STZ mice, as represented by increased UACR, EMT, foot process fusion and basement membrane thickening (Fig. 5A, E–H). However, little difference was observed in serum UN, CR and Cys-c between the two groups (Fig. 5B–D).

Taken together, the appearance of proteinuria, foot process fusion, EMT or apoptosis of podocytes suggested disrupted integrity of glomerular filtration barrier in MAP4 KI mice reminiscent of the typical DN pathology, and STZ treatment worsen such DN pathology in MAP4 KI mice as compared to WT littermates. Simultaneous occurrence of retinopathy in MAP4 KI mice suggested the nephropathy may attribute to the onset of microvascular disease in diabetes.

### MAP4 phosphorylation induced EMT, cytoskeleton rearrangement and apoptosis in podocytes

**Fig. 6:**
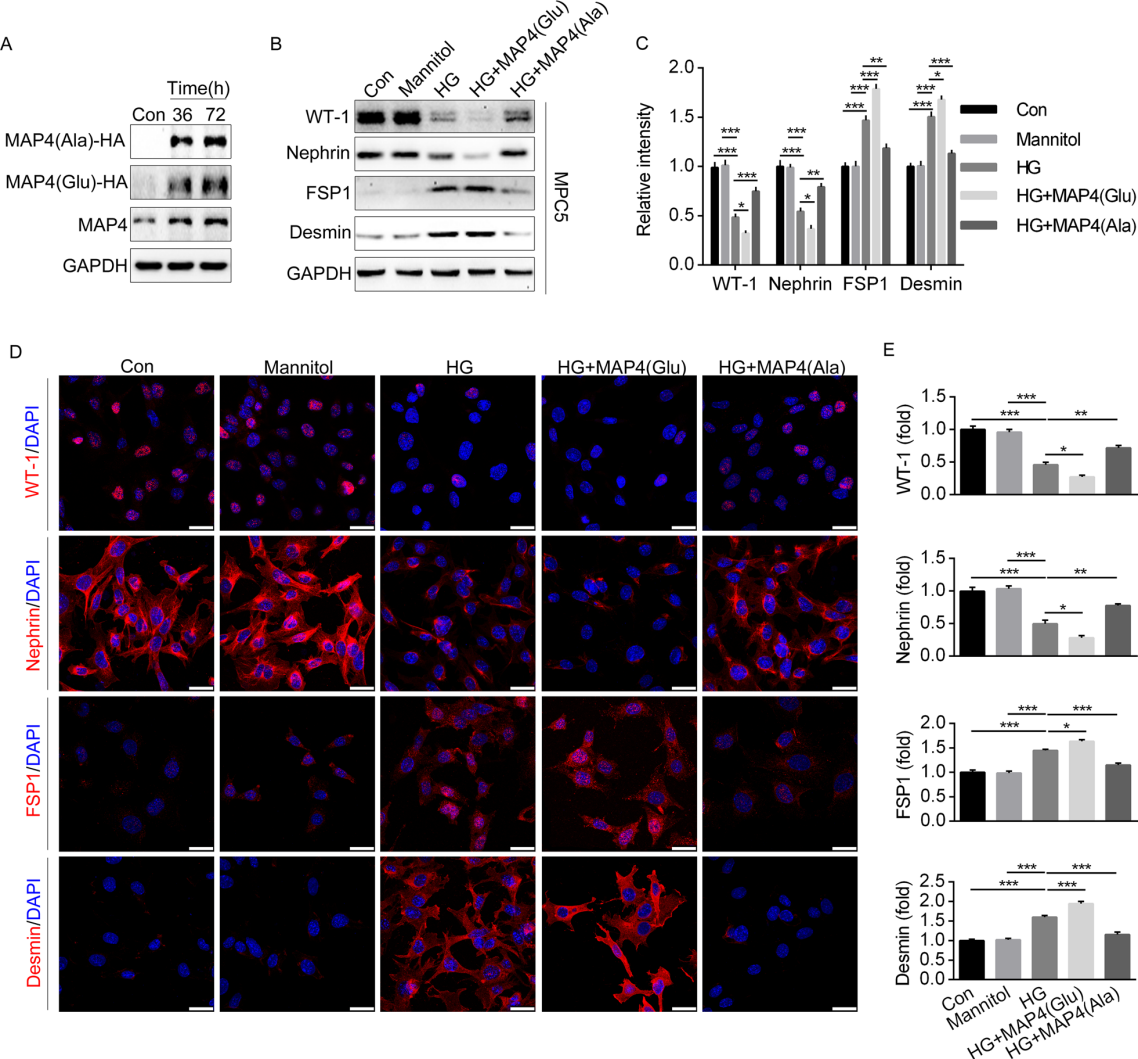
MAP4 phosphorylation induced EMT in podocyte. **A** Identification of adenovirus efficacy of MAP4(Ala) or MAP4(Glu). **B**, **C** Representative WB (**B**) and quantitative analysis (**C**) showing the epithelial and mesenchymal cell markers of podocyte after MAP4(Ala) or MAP4(Glu) transfection with or without HG stimulation. **D**, **E** Representative confocal immunofluorescence images (**D**) and quantitative analysis (**E**) showing the epithelial and mesenchymal cell markers of podocyte after MAP4(Ala) or MAP4(Glu) transfection with or without HG stimulation. Bar, 25 µm. MPC5, cell line of mouse podocyte. Data were shown as mean ± SEM. The experiment was conducted 6 times. *P < 0.05, **P < 0.01, ***P < 0.001.* P* values were derived from one-way ANOVA with Bonferroni’s post-test

Earlier evidence suggested that podocytes may play an essential role in defective glomerular filtration barrier triggered by MAP4 hyperphosphorylation. To discern the impact of MAP4 phosphorylation in podocytes, adenovirus carrying MAP4(Ala) or MAP4(Glu) was applied to podocytes under high glucose (HG) challenge. WB analysis was performed to confirm MAP4(Glu) or MAP4(Ala) transfection (Fig. 6A). HG treatment down-regulated WT-1 and Nephrin, while up-regulating FSP1 and Desmin. Transfection with MAP4(Glu) and MAP4(Ala) aggravated and alleviated HG-induced dedifferentiation in podocytes, respectively (Fig. 6B, C). Immunofluorescence staining revealed similar trend for WT-1, Nephrin, FSP1 and Desmin in podocytes (Fig. 6D, E).

**Fig. 7:**
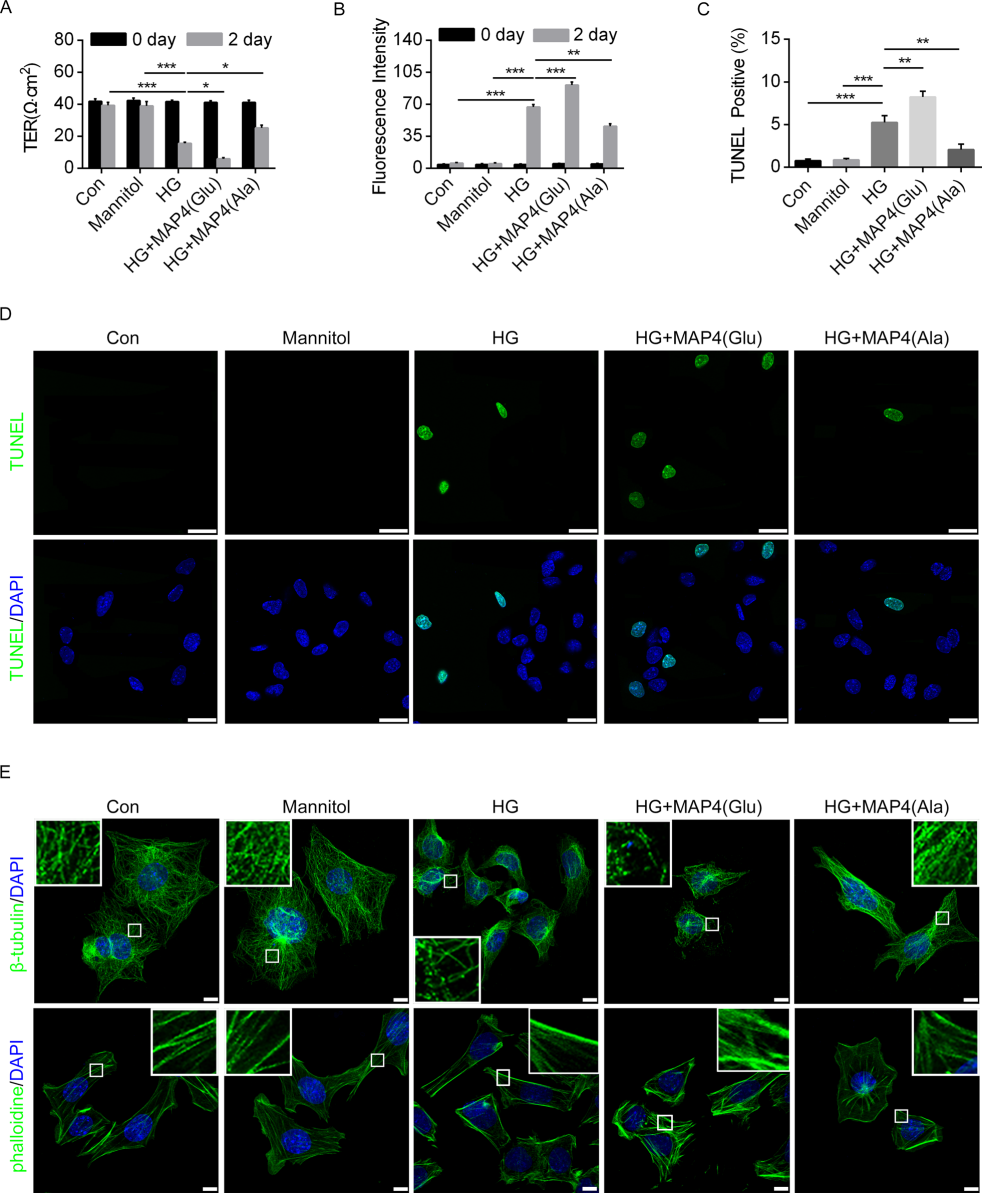
MAP4 phosphorylation induced cytoskeleton rearrangement and apoptosis in podocyte. **A**, **B** The permeability of podocyte was assessed by measuring the influx of FITC-conjugated dextran and the TER across the cells. **C**, **D** Podocyte apoptosis was detected by TUNEL staining. Bar, 25 µm. **E** Representative confocal immunofluorescence images showing the MTs and F-actin organization of the podocytes after MAP4(Ala) or MAP4(Glu) transfection with or without HG stimulation. Bar, 10 μm. The inserts showed high magnification images of the MT and F-actin network. Data were shown as mean ± SEM. The experiment was conducted 6 times. *P < 0.05, **P < 0.01, ***P < 0.001.* P* values were derived from one-way ANOVA with Bonferroni’s post-test

The effect of MAP4 phosphorylation on barrier function of podocytes was also examined. Permeability of monolayer podocyte was monitored by evaluating the TER or FITC-conjugated dextran influx across cells. HG treatment induced decreased TER while increased dextran leakage. MAP4(Ala) transfection was found to abolish HG-induced hyperpermeability, resulting in a 1.46-fold decrease in dextran leakage, and a 1.62-fold elevation in TER as compared to HG treatment group. To the contrary, transduction with MAP4(Glu) resulted in higher hyperpermeability (1.36-fold elevation in dextran leakage and 2.58-fold decrease in TER as compared to HG group) (Fig. 7A, B). Using TUNEL staining, a rise in apoptosis was detected in HG group, the effect of which was ameliorated and accentuated by MAP4(Ala) and MAP4(Glu) transfection, respectively (Fig. 7C, D). Given the role of MAP4 in MT dynamic regulation, and that cytoskeleton possesses an essential effect in maintaining the integrity of podocytes foot processes, we determined whether MAP4 phosphorylation regulates permeability though cytoskeleton rearrangement. Scrutiny of F-actin and MT distribution in podocytes revealed that HG treatment induced the depolymerization of MT, the stress fiber reorganization of F-actin, and the re-distribution of F-actin from the perinuclear region towards the periphery of cells. MAP4(Glu) transfection accentuated while MAP4(Ala) transfection mitigated these responses (Fig. 7E).

### p38/MAPK activation mediated MAP4 phosphorylation induced podocyte EMT and apoptosis

**Fig. 8:**
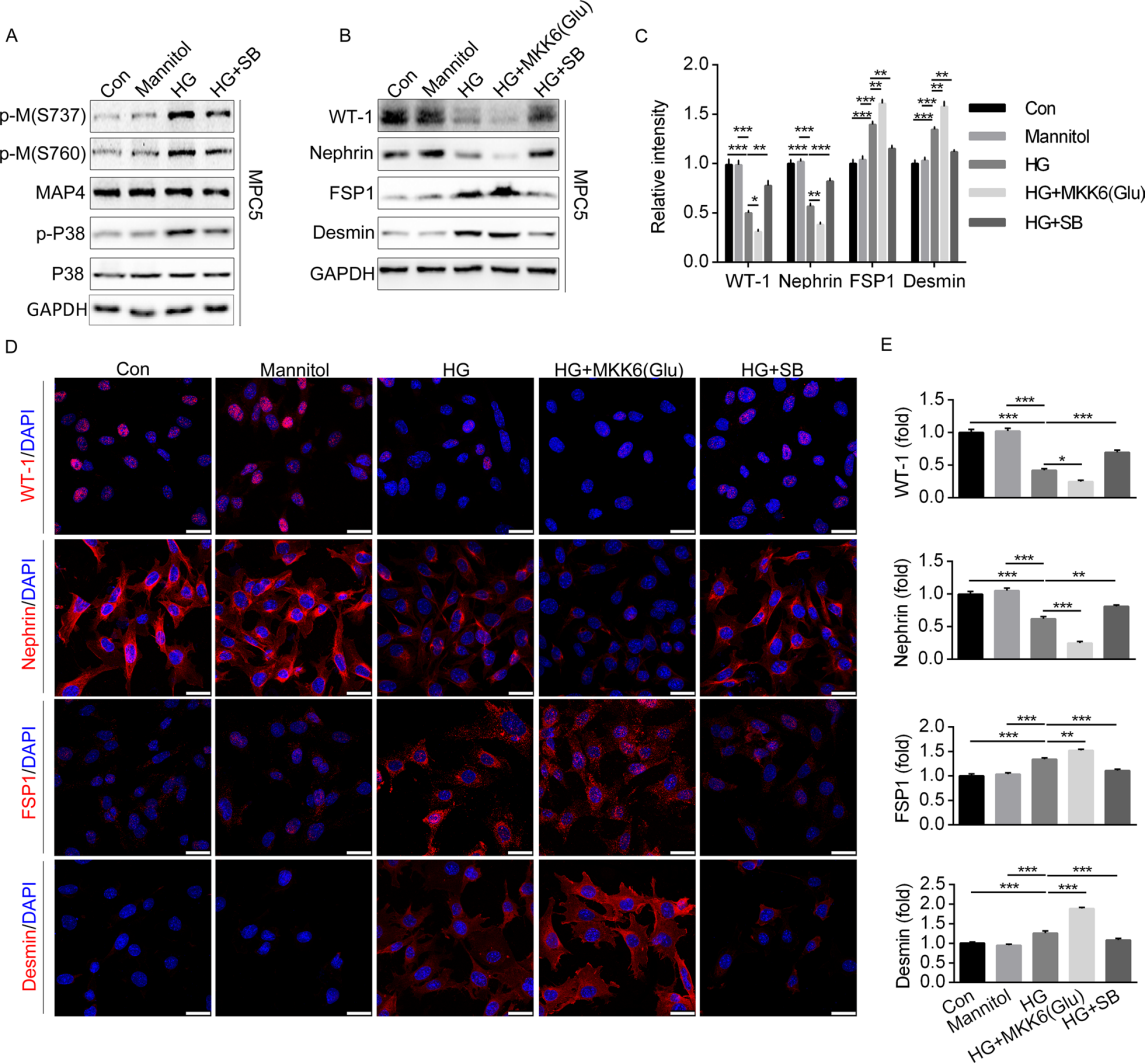
p38/MAPK activation mediated HG-induced podocyte EMT. **A** Detection of p-M (S737 and S760) and p-P38 with or without SB203580 (SB) and MKK6(Glu) transfection. **B**, **C** WB analysis showing the epithelial and mesenchymal cell markers of podocyte with or without SB or MKK6(Glu) transfection. **D**, **E** Representative confocal immunofluorescence images (**D**) and quantitative analysis (**E**) showing the epithelial and mesenchymal cell markers of podocyte. Bar, 25 µm. Data were shown as mean ± SEM. The experiment was conducted 6 times. *P < 0.05, **P < 0.01, ***P < 0.001.* P* values were derived from one-way ANOVA with Bonferroni’s post-test

Our previous study indicated that p38/MAPK serves as a crucial kinase modulating MAP4 phosphorylation in hypoxic cardiomyocytes [17]. Here we assessed whether p38/MAPK was involved in HG-induced MAP4 phosphorylation in podocytes. Our findings revealed elevated expressions of phosphorylation of MAP4 (S737 and S760) and P38 following HG stimulation (without affecting MAP4 and p38). In contrast, SB203580 (a p38/MAPK inhibitor) suppressed HG-induced p38/MAPK and MAP4 phosphorylation (S737 and S760) (Fig. 8A and Additional file 1: Fig. S1). These findings inferred that p38/MAPK was a key upstream kinase to promote phosphorylated MAP4 under HG challenge.

**Fig. 9:**
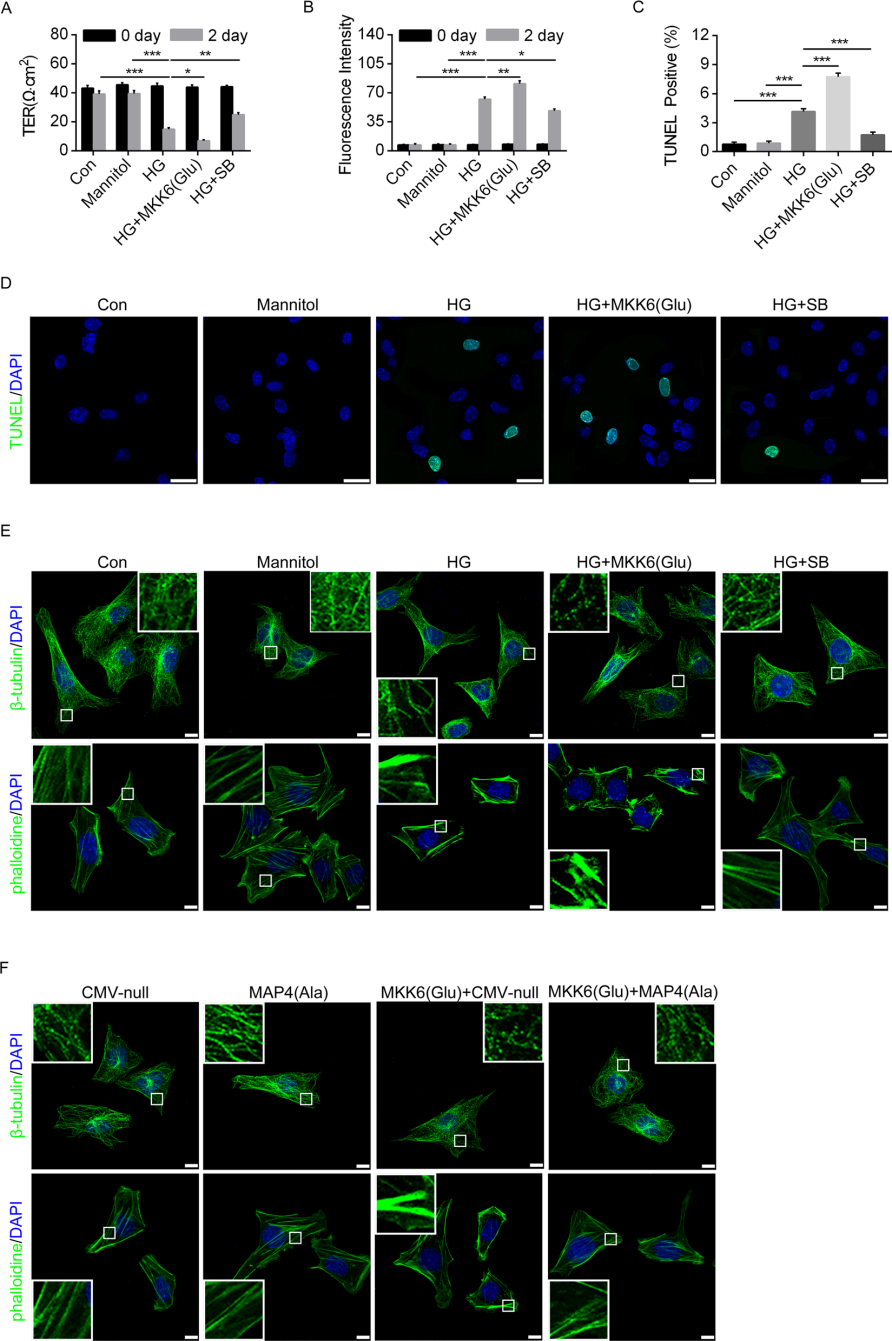
p38/MAPK activation mediated HG-induced podocyte apoptosis and cytoskeleton. **A**, **B** The permeability of podocyte was assessed by measuring the influx of FITC-conjugated dextran and the TER across the cells. **C**, **D** TUNEL staining showed podocyte apoptosis. Bar, 25 µm. **E** Representative confocal immunofluorescence images showing the MTs and F-actin organization of the podocytes. Bar, 10 μm. The inserts showed high magnification images of the MT and F-actin network. **F** Representative immunofluorescence images showing the MTs and F-actin organization of the podocyte after the cells were transiently transfected with MAP4(Ala), MKK6(Glu) or both. Bar, 10 μm. The inserts showed high magnification images of the peripheral MT network. Data were shown as mean ± SEM. The experiment was conducted 6 times. *P < 0.05, **P < 0.01, ***P < 0.001.* P* values were derived from one-way ANOVA with Bonferroni’s post-test

We next discern the effect of p38/MAPK in HG-mediated podocyte EMT, hyperpermeability and apoptosis. Podocytes were transfected with MKK6(Glu) adenovirus to activate p38/MAPK, while pretreatment of SB203580 inhibited HG-induced p38/MAPK activation. WB analysis indicated that MKK6(Glu) aggravated while SB203580 alleviated HG-induced reduction of epithelial markers and elevation of mesenchymal markers in podocytes (Fig. 8B, C). Immunofluorescence staining of podocytes revealed similar pattern in WT-1, Nephrin, FSP1 and Desmin (Fig. 8D, E). In addition, SB203580 pretreatment protected against HG-induced podocytes hyperpermeability and apoptosis, while the problems were aggravated after MKK6(Glu) transfection (Fig. 9A–D). Furthermore, MKK6(Glu) transfection accentuated while SB203580 mitigated HG-induced changes in cytoskeleton (Fig. 9E).

Then we assessed the effect of MAP4 phosphorylation in p38/MAPK-mediated cytoskeleton rearrangement. Podocytes were transfected with MKK6(Glu), MAP4(Ala) or both. Our data revealed that podocytes overexpressing MAP4(Ala) were more resistant to MKK6(Glu) transfection compared with the CMV-null control on cytoskeleton rearrangement (Fig. 9F). These data denoted the role of p38/MAPK as an upstream regulator for MAP4 phosphorylation and further demonstrated a crucial role of MAP4 phosphorylation in p38/MAPK-induced podocyte EMT, hyperpermeability and apoptosis.

## Discussion

Salient findings from current study indicated that MAP4, the classical skeletal protein, was phosphorylated in the urine of diabetic patients and the kidney of the STZ-induced diabetic mice, which was accompanied with glomerular proteinuria associated with nephropathy. The MAP4 KI mice, a mouse model of hyperphosphorylated MAP4 at S737 and S760, triggered proteinuria with aging, accompanied with increased EMT and apoptosis of podocytes. Additionally, MAP4 KI mice were much more susceptible to STZ treatment and showed robust DN pathology as compared to WT mice. In cultured podocytes, MAP4 phosphorylation was universal increased following hyperglycemia as the down-stream effector of p38/MAPK, which led to podocytes undergoing functional, morphological, and molecular changes reminiscent of apoptosis and EMT through induced cytoskeleton rearrangement. Otherwise, it should be indicated that the MAP4 KI mice also developed retinopathy. The diabetic retinopathy and DN are the main microvascular complications of diabetes, and the co-occurrence of retinopathy and nephropathy in MAP4 KI mice suggest that the MAP4 phosphorylation may act as the common mechanism mediating the microvascular disease in diabetes.

In current study, we firstly figured out that MAP4 and phosphorylated MAP4 could be detected in urine, indicating MAP4 was a potential secretory protein. However, a key question was that which cell in urinary system secreted MAP4? Based on our recent research, we thought endothelial cell might be the cell. In detail, this article revealed that NLRP3 related pyroptosis was activated by MAP4 phosphorylation in endothelial cells [13]. During the process, a pore was formed on cell membrane and contributed to the release of inflammatory molecules including IL-18 and IL-1β. Whether this pore also functioned as the channel of MAP4 secretion was uncertain and required more efforts.

Podocyte loss and dysfunction are known to possess a crucial effect in the pathogenesis of proteinuria and glomerulosclerosis [5]. Podocytes are terminally differentiated cells which cover GBM over glomerular capillary. Podocytes maintain glomerular structural integrity through GBM counteracting outward expansion, for which the foot processes is the major functional unit [15]. When subject to pathological challenge, podocytes undergo a series of changes, involving EMT, dissociation, hypertrophy, and apoptosis, relying on the degree of injury [7, 26, 34]. Since their limited ability of proliferation, podocyte dissociation from GBM and apoptosis will unavoidably cause cell loss or drop out, which decrease density of podocyte, leading to impaired glomerular filtration, and ultimately proteinuria. Recent experimental data demonstrates that podocyte loss often occurs in late stage of chronic kidney disease in which proteinuria is already distinct [6, 11]. It is thus plausible to infer that the abnormal modulation of podocyte function or differentiation, rather than podocyte loss, might be a trigger of proteinuria in various clinical conditions.

EMT of podocytes is characterized by deficiency of epithelial markers, including P-cadherin, ZO-1, and Nephrin, and de novo gain classic mesenchymal markers, such as collagen I, FSP1, Desmin and fibronectin [22]. As the crucial part of the slit diaphragm cell adhesion complexes, loss of Nephrin will contribute to podocyte dissociation from GBM and disrupt the integrity of slit diaphragm [2]. EMT has recently been observed in STZ-treated diabetic rats [9], and exists when subjected to hypoxia, reactive oxygen species, advanced glycation end products and numerous pro-fibrotic cytokines, growth factors and metalloproteinases [35].

One interesting finding from the present study is the profound apoptosis and EMT of podocytes in response to MAP4 hyperphosphorylation in STZ-treated and MAP4 KI mice. An essential feature that distinguishes podocytes from other renal cells is that they are terminally differentiated, and have no proliferative ability when subject to injury or noxious stimuli. Cell proliferation rate seen in our study exhibited little difference among WT, STZ-treated and MAP4 KI mice, which suggested that increased mesenchymal cells were possibly attributed to the development of podocyte EMT rather than mesangial cell proliferation. In spite of this, many questions remain unknown such as if increased mesenchymal cells originated from development EMT of endothelial cells, whether proteinuria was also attributed to overt apoptosis or EMT of podocytes.

Cytoskeleton rearrangement is crucial to accomplish EMT. It is not only the default consequence of EMT activation, but also a regulator in this process [25, 32]. It has been reported that actin reorganization is tightly connected to foot processes effacement related to proteinuria [18]. However, the precise mechanism regulating the cytoskeleton rearrangement when podocytes undergo EMT is still unclear and needs further investigation. Interactions between MT and actin represent a phenomenon governing a number of basic cellular processes, such as cell motility and cell division. MAPs have been shown to be involved in regulating the interactions of MT and actin in the formation of cytoskeletal network [12]. In this study, we noted overtly elevated phosphorylation of MAP4 in STZ-induced diabetic mice. On the other hand, mutated mouse model with hyperphosphorylated MAP4 (S737E and S760E) displayed proteinuria with aging, accompanied with prominent EMT of podocytes, in a manner reminiscent of the pathologic changes in the DN, furthermore, STZ stimulation aggravated such kidney pathological changes in MAP4 KI mice as compared to WT littermates. It is an interesting phenomenon in the present study that both aging and STZ treatment are involved in DN in our present study. In MAP4 KI- and STZ-induced mice, nephropathy progress overtime is the result of accumulated damage induced by MAP4 hyperphosphorylation or high glucose. Age is not the direct cause, but aged organ/cells have less compensating ability. Thus, we observed worse diabetic nephropathy phenotype in aged MAP4 KI mice. In all, aging and accumulated damages induced by MAP4 hyperphosphorylation and high glucose are both conditions making MAP4 KI mice harder to keep their in vivo homeostasis. In addition, in vitro data revealed that HG promoted MAP4 hyperphosphorylation, and either HG or aberrant regulation of MAP4 phosphorylation led to cytoskeleton and F-actin rearrangement and impaired filtration barrier function in podocytes. MAP4(Ala) mutant, which mimicked dephosphorylated MAP4, suppressed EMT and alleviated increased cell permeability. Therefore, these in vivo and in vitro observations favored a novel insight for MAP4 in modulating podocyte EMT and proteinuria in the development of DN through MAP4 phosphorylation.

In our previous study, p38/MAPK activation was sufficient to trigger MAP4 phosphorylation in cardiomyocytes [16]. Among many factors implicated in the pathogenesis of DN, p38/MAPK served as one of critical mediators for inflammatory reactions and mitochondrial malfunction in diabetes [27]. Activated p38/MAPK was found in renal proximal tubular epithelial cells (PTECs) and contributed to EMT [37]. Aberrant p38 phosphorylation was also correlated with podocyte cytoskeletal dynamics [18]. In our hands, inhibition of p38/MAPK was proved to alleviate MT, F-actin arrangement and hyperpermeability of podocytes in response to HG challenge accompanied with decreased MAP4 phosphorylation. Our data are the first, to our best knowledge, to suggest a novel role for p38/MAPK in regulating podocyte EMT through phosphorylating MAP4 in DN. Our earlier studies depicted a role for MAP4 phosphorylation in endogenous apoptosis in cardiomyocytes in response to hypoxia. Whether phosphorylated MAP4 mediates podocyte apoptosis through similar mechanism warrants further scrutiny.

## Conclusions

Here in the present study using a hyperphosphorylated MAP4 mouse strain, we showed that MAP4 phosphorylation induced MT and F-actin rearrangement, podocyte EMT and apoptosis, leading to proteinuria, in a manner reminiscent of DN. Blockade of p38/MAPK signaling prevented the dedifferentiation and apoptosis of podocytes. These data implied that manipulating p38/MAPK-MAP4 phosphorylation signaling might unearth a future therapeutic target to alleviate proteinuria and renal fibrosis in DN.

## Supplementary Information

**Additional file 1. **The quantification of p-M (S737 and S760) and p-P38 with or without SB203580 (SB) and MKK6 (Glu) transfection. ***p < 0.001.

## Data Availability

Not applicable.

## Abbreviations

DN: Diabetic nephropathy
MAP4: Microtubule associated protein 4
p-MAP4: MAP4 phosphorylation
STZ: Streptozocin
EMT: Epithelial-to-mesenchymal transition
HG: High glucose
MAPs: Microtubule-associated proteins
MT: Microtubule
MKK6: Mitogen-activated protein kinase 6
MAP4 KI: MAP4 (S737/760E) knock-in
CR: Urine creatinine
UN: Urea nitrogen
Cys-c: Cystatin c
TEM: Transmission electron microscope
GBM: Glomerular basement membrane
WT-1: Wilms Tumor protein
FSP1: Fibroblast-specific protein 1
TUNEL: Terminal deoxynucleotidyl transferase-mediated dUTP nick-end labeling
EdU: 5-Ethynyl-2'-deoxyuridine
ELISA: Enzyme-Linked Immunosorbent Assay
TER: Measurement of transepithelial electric resistance
FITC: Fluorescein isothiocyanate
ANOVA: One-way analysis of variance
PTECs: Proximal tubular epithelial cells

## References

[1] Asanuma K, Kim K, Oh J, Giardino L, Chabanis S, Faul C (2005). Synaptopodin regulates the actin-bundling activity of alpha-actinin in an isoform-specific manner. J Clin Investig.

[2] Asanuma K, Mundel P (2003). The role of podocytes in glomerular pathobiology. Clin Exp Nephrol.

[3] Chen W, Jiang Y, Han J, Hu J, He T, Yan T (2017). Atgl deficiency induces podocyte apoptosis and leads to glomerular filtration barrier damage. FEBS J.

[4] Czajka A, Ajaz S, Gnudi L, Parsade CK, Jones P, Reid F (2015). Altered mitochondrial function, mitochondrial DNA and reduced metabolic flexibility in patients with diabetic nephropathy. EBioMedicine.

[5] Daehn I, Casalena G, Zhang T, Shi S, Fenninger F, Barasch N (2014). Endothelial mitochondrial oxidative stress determines podocyte depletion in segmental glomerulosclerosis. J Clin Investig.

[6] Dai C, Stolz DB, Bastacky SI, St-Arnaud R, Wu C, Dedhar S (2006). Essential role of integrin-linked kinase in podocyte biology: Bridging the integrin and slit diaphragm signaling. J Am Soc Nephrol.

[7] Dai H, Liu Q (2017). Research progress on mechanism of podocyte depletion in diabetic nephropathy. J Diabetes Res.

[8] Drewes G, Ebneth A, Mandelkow EM (1998). MAPs, MARKs and microtubule dynamics. Trends Biochem Sci.

[9] Du L, Hao M, Li C, Wu W, Wang W, Ma Z (2017). Quercetin inhibited epithelial mesenchymal transition in diabetic rats, high-glucose-cultured lens, and SRA01/04 cells through transforming growth factor-beta2/phosphoinositide 3-kinase/Akt pathway. Mol Cell Endocrinol.

[10] Ebneth A, Drewes G, Mandelkow EM, Mandelkow E (1999). Phosphorylation of MAP2c and MAP4 by MARK kinases leads to the destabilization of microtubules in cells. Cell Motil Cytoskeleton.

[11] El-Aouni C, Herbach N, Blattner SM, Henger A, Rastaldi MP, Jarad G (2006). Podocyte-specific deletion of integrin-linked kinase results in severe glomerular basement membrane alterations and progressive glomerulosclerosis. J Am Soc Nephrol.

[12] Farias GA, Munoz JP, Garrido J, Maccioni RB (2002). Tubulin, actin, and tau protein interactions and the study of their macromolecular assemblies. J Cell Biochem.

[13] Feng Y, Li L, Zhang Q, Zhang J, Huang Y (2021). Microtubule associated protein 4 (MAP4) phosphorylation reduces cardiac microvascular density through NLRP3-related pyroptosis. Cell Death Discov.

[14] Gallagher H, Suckling RJ (2016). Diabetic nephropathy: where are we on the journey from pathophysiology to treatment?. Diabetes Obes Metab.

[15] Garg P (2018). A review of podocyte biology. Am J Nephrol.

[16] Hu J, Chu Z, Han J, Zhang Q, Zhang D, Dang Y (2014). Phosphorylation-dependent mitochondrial translocation of MAP4 is an early step in hypoxia-induced apoptosis in cardiomyocytes. Cell Death Dis.

[17] Hu JY, Chu ZG, Han J, Dang YM, Yan H, Zhang Q (2010). The p38/MAPK pathway regulates microtubule polymerization through phosphorylation of MAP4 and Op18 in hypoxic cells. Cell Mol Life Sci.

[18] Hu M, Fan M, Zhen J, Lin J, Wang Q, Lv Z (2017). FAK contributes to proteinuria in hypercholesterolaemic rats and modulates podocyte F-actin re-organization via activating p38 in response to ox-LDL. J Cell Mol Med.

[19] Illenberger S, Drewes G, Trinczek B, Biernat J, Meyer HE, Olmsted JB (1996). Phosphorylation of microtubule-associated proteins MAP2 and MAP4 by the protein kinase p110mark. Phosphorylation sites and regulation of microtubule dynamics. J Biol Chem.

[20] Kang YS, Li Y, Dai C, Kiss LP, Wu C, Liu Y (2010). Inhibition of integrin-linked kinase blocks podocyte epithelial-mesenchymal transition and ameliorates proteinuria. Kidney Int.

[21] Kitazawa H, Iida J, Uchida A, Haino-Fukushima K, Itoh TJ, Hotani H (2000). Ser787 in the proline-rich region of human MAP4 is a critical phosphorylation site that reduces its activity to promote tubulin polymerization. Cell Struct Funct.

[22] Kumar PA, Welsh GI, Raghu G, Menon RK, Saleem MA, Reddy GB (2016). Carboxymethyl lysine induces EMT in podocytes through transcription factor ZEB2: Implications for podocyte depletion and proteinuria in diabetes mellitus. Arch Biochem Biophys.

[23] Li L, Hu J, He T, Zhang Q, Yang X, Lan X (2015). P38/MAPK contributes to endothelial barrier dysfunction via MAP4 phosphorylation-dependent microtubule disassembly in inflammation-induced acute lung injury. Sci Rep.

[24] Li L, Zhang Q, Zhang X, Zhang J, Wang X, Ren J (2018). Microtubule associated protein 4 phosphorylation leads to pathological cardiac remodeling in mice. EBioMedicine.

[25] Li N, Jiang P, Du W, Wu Z, Li C, Qiao M (2011). Siva1 suppresses epithelial-mesenchymal transition and metastasis of tumor cells by inhibiting stathmin and stabilizing microtubules. Proc Natl Acad Sci USA.

[26] Ling L, Tan Z, Zhang C, Gui S, Hu Y, Chen L (2018). Long noncoding RNA ENSRNOG00000037522 is involved in the podocyte epithelialmesenchymal transition in diabetic rats. Int J Mol Med.

[27] Liu WT, Peng FF, Li HY, Chen XW, Gong WQ, Chen WJ (2016). Metadherin facilitates podocyte apoptosis in diabetic nephropathy. Cell Death Dis.

[28] Locatelli F, Pozzoni P, Del Vecchio L (2004). Renal replacement therapy in patients with diabetes and end-stage renal disease. J Am Soc Nephrol.

[29] Najafian B, Basgen JM, Mauer M (2002). Estimating mean glomerular volume using two arbitrary parallel sections. J Am Soc Nephrol.

[30] Raingeaud J, Whitmarsh AJ, Barrett T, Derijard B, Davis RJ (1996). MKK3- and MKK6-regulated gene expression is mediated by the p38 mitogen-activated protein kinase signal transduction pathway. Mol Cell Biol.

[31] Rask-Madsen C, King GL (2010). Diabetes: podocytes lose their footing. Nature.

[32] Shankar J, Messenberg A, Chan J, Underhill TM, Foster LJ, Nabi IR (2010). Pseudopodial actin dynamics control epithelial-mesenchymal transition in metastatic cancer cells. Cancer Res.

[33] Shankland SJ, Pippin JW, Reiser J, Mundel P (2007). Podocytes in culture: past, present, and future. Kidney Int.

[34] Wang RM, Wang ZB, Wang Y, Liu WY, Li Y, Tong LC (2018). Swiprosin-1 promotes mitochondria-dependent apoptosis of glomerular podocytes via P38 MAPK pathway in early-stage diabetic nephropathy. Cell Physiol Biochem.

[35] Wei SC, Yang J (2016). Forcing through tumor metastasis: the interplay between tissue rigidity and epithelial-mesenchymal transition. Trends Cell Biol.

[36] Ying Q, Wu G (2017). Molecular mechanisms involved in podocyte EMT and concomitant diabetic kidney diseases: an update. Ren Fail.

[37] Zhang X, Liang D, Chi ZH, Chu Q, Zhao C, Ma RZ (2015). Effect of zinc on high glucose-induced epithelial-to-mesenchymal transition in renal tubular epithelial cells. Int J Mol Med.

[38] Zurbig P, Jerums G, Hovind P, Macisaac RJ, Mischak H, Nielsen SE (2012). Urinary proteomics for early diagnosis in diabetic nephropathy. Diabetes.

